# Crystal structure of *catena*-poly[hemi[1,3-bis­(2,6-diisoprop­ylphenyl)imidazolium] [[μ_3_-acetato-κ^3^ *O*:*O*:*O*′-tri-μ_2_-acetato-κ^6^ *O*:*O*′-dicopper(II)(*Cu*—*Cu*)]-μ-chlorido] di­chloro­methane sesqui­solvate]

**DOI:** 10.1107/S2056989015013675

**Published:** 2015-07-25

**Authors:** Mohammad Iqbal, James Raftery, Peter Quayle

**Affiliations:** a School of Chemistry, University of Manchester, Manchester M13 9PL, UK; b Pakistan Institute of Nuclear Science and Technology, PO Box Nilore, Islamabad, Pakistan

**Keywords:** crystal structure, coordination polymer, copper(II) tetra­acetate, paddle-wheel, imidazolium, paramagnetism

## Abstract

The title copper(II) complex, {(C_27_H_37_N_2_)[Cu_4_(CH_3_COO)_8_Cl]·3CH_2_Cl_2_}_*n*_, is a one-dimensional coordination polymer. The asymmetric unit is composed of a copper(II) tetra­acetate paddle-wheel complex, a Cl^−^ anion situated on a twofold rotation axis, half a 1,3-bis­(2,6-diisoprop­ylphenyl)imidazolium cation (the whole mol­ecule being generated by twofold rotation symmetry) and one and a half of a di­chloro­methane solvent mol­ecule (one being located about a twofold rotation axis). The central metal-organic framework comprises of a tetra­nuclear copper(II) acetate ‘paddle-wheel’ complex which arises from the dimerization of the copper(II) tetra­acetate core comprising of three μ_2_-bidentate acetate and one μ_3_-tridentate acetate ligands per binuclear paddle-wheel complex. Both Cu^II^ atoms of the binuclear component adopt a distorted square-pyramidal coordination geometry (τ = 0.04), with a Cu⋯Cu separation of 2.6016 (2) Å. The apical coordination site of one Cu^II^ atom is occupied by an O atom of a neighbouring acetate bridge [Cu—O = 2.200 (2) Å], while that of the second Cu^II^ atom is occupied by a bridging chloride ligand [Cu⋯Cl = 2.4364 (4) Å]. The chloride bridge is slightly bent with respect to the Cu⋯Cu inter­nuclear axis [Cu—Cl—Cu = 167.06 (6)°] and the tetra­nuclear units are located about a twofold rotation axis, forming the one-dimensional polymer that propagates along [101]. Charge neutrality is maintained by the inclusion of the 1,3-bis­(2,6-diisoprop­ylphenyl)imidazolium cation within the crystal lattice. In the crystal, the cation and di­chloro­methane solvent mol­ecules are linked to the coordin­ation polymer by various C—H⋯O and C—H⋯Cl hydrogen bonds. There are no other significant inter­molecular inter­actions present.

## Related literature   

For the use of N-heterocyclic carbenes (NHCs) as ancillary ligands for the preparation of transition-metal-based catalysts, see: Hopkinson *et al.* (2014 ▸). For their use in organic transformations, see: Faulkner *et al.* (2005 ▸); Bull *et al.* (2008 ▸). For details of the magnetic properties of binuclear Cu^II^ carboxyl­ate compounds, see: Kato *et al.* (1964 ▸); Zhang *et al.* (2005 ▸); Cotton *et al.* (2000 ▸), and for their electrochemical behaviour, see: Paschke *et al.* (2003 ▸). For examples of copper(II) paddle-wheel structures, see: de Meester *et al.* (1973 ▸); Ackermann *et al.* (2000 ▸). For chloride-bridged binuclear systems, see: Chen *et al.* (2015 ▸). For imidazolium-functionalized acetate ligands, see: Suresh *et al.* (2015 ▸). For the description of the fivefold coordination symmetry parameter, τ, see: Addison *et al.* (1984 ▸).
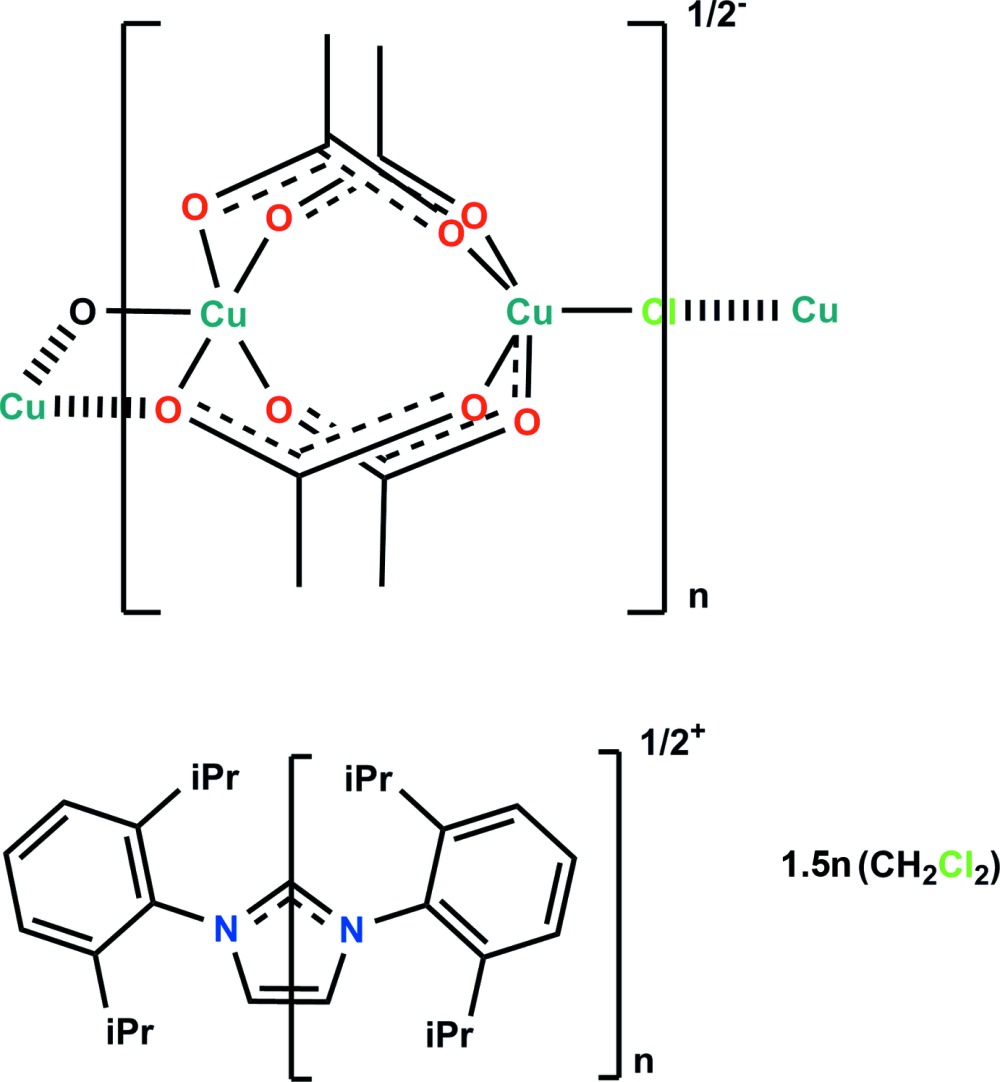


## Experimental   

### Crystal data  

(C_27_H_37_N_2_)[Cu_4_(C_2_H_3_O_2_)_8_Cl]·3CH_2_Cl_2_
*M* *_r_* = 1406.32
Monoclinic, 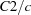

*a* = 22.097 (2) Å
*b* = 13.146 (2) Å
*c* = 23.607 (3) Å
β = 117.122 (4)°
*V* = 6103.5 (13) Å^3^
*Z* = 4
Mo *K*α radiation
μ = 1.74 mm^−1^
*T* = 100 K
0.22 × 0.13 × 0.05 mm

### Data collection  

Bruker SMART CCD area-detector diffractometer
Absorption correction: multi-scan (*SADABS*; Bruker, 2001 ▸) *T*_min_ = 0.700, *T*_max_ = 0.918
26111 measured reflections
7274 independent reflections
5215 reflections with *I* > 2σ(*I*)
*R*_int_ = 0.079

### Refinement  

*R*[*F* ^2^ > 2σ(*F* ^2^)] = 0.054
*wR*(*F* ^2^) = 0.098
*S* = 0.98
7274 reflections
348 parameters
H-atom parameters constrained
Δρ_max_ = 0.70 e Å^−3^
Δρ_min_ = −0.46 e Å^−3^

### 

Data collection: *SMART* (Bruker, 2001 ▸); cell refinement: *SAINT* (Bruker, 2001 ▸); data reduction: *SAINT*; program(s) used to solve structure: *SHELXS97* (Sheldrick, 2008 ▸); program(s) used to refine structure: *SHELXL2014* (Sheldrick, 2015 ▸); molecular graphics: *PLATON* (Spek, 2009 ▸); software used to prepare material for publication: *SHELXL2014* and *PLATON*.

## Supplementary Material

Crystal structure: contains datablock(s) I, global. DOI: 10.1107/S2056989015013675/su5152sup1.cif

Structure factors: contains datablock(s) I. DOI: 10.1107/S2056989015013675/su5152Isup2.hkl

**Fig. 1.**
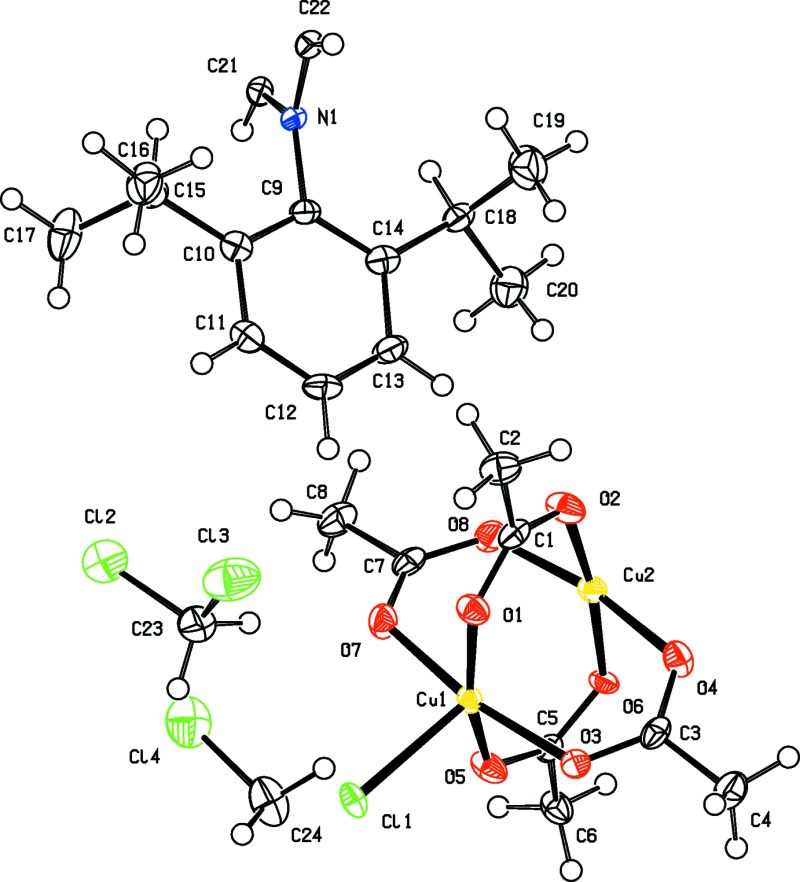
A view of the molecular structure of the asymmetric unit of the title compound. Displacement ellipsoids are drawn at the 50% probability level.

. DOI: 10.1107/S2056989015013675/su5152fig1.tif
A view of the mol­ecular structure of the asymmetric unit of the title compound. Displacement ellipsoids are drawn at the 50% probability level.

**Fig. 2.**
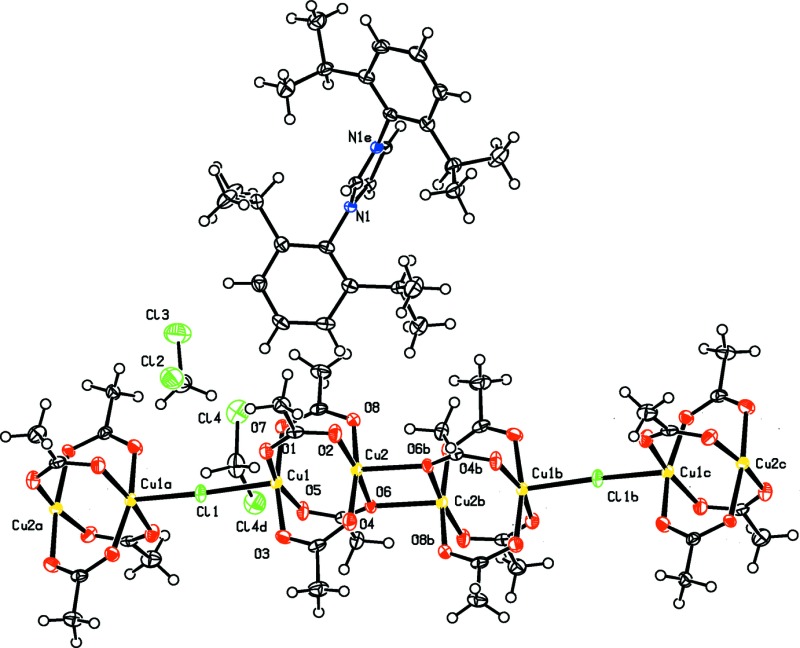
A view of the tetranuclear paddle-wheel unit of the title polymeric compound [symmetry codes: (a) -x, y, -z + 1/2; (b) -x + 1/2, -y + 1/2, -z + 1; (c) x + 1/2, -y + 1/2, z + 1/2; (d) -x, y, -z + 1/2; (e) -x + 1, y, -z + 1/2].

**Fig. 3.**
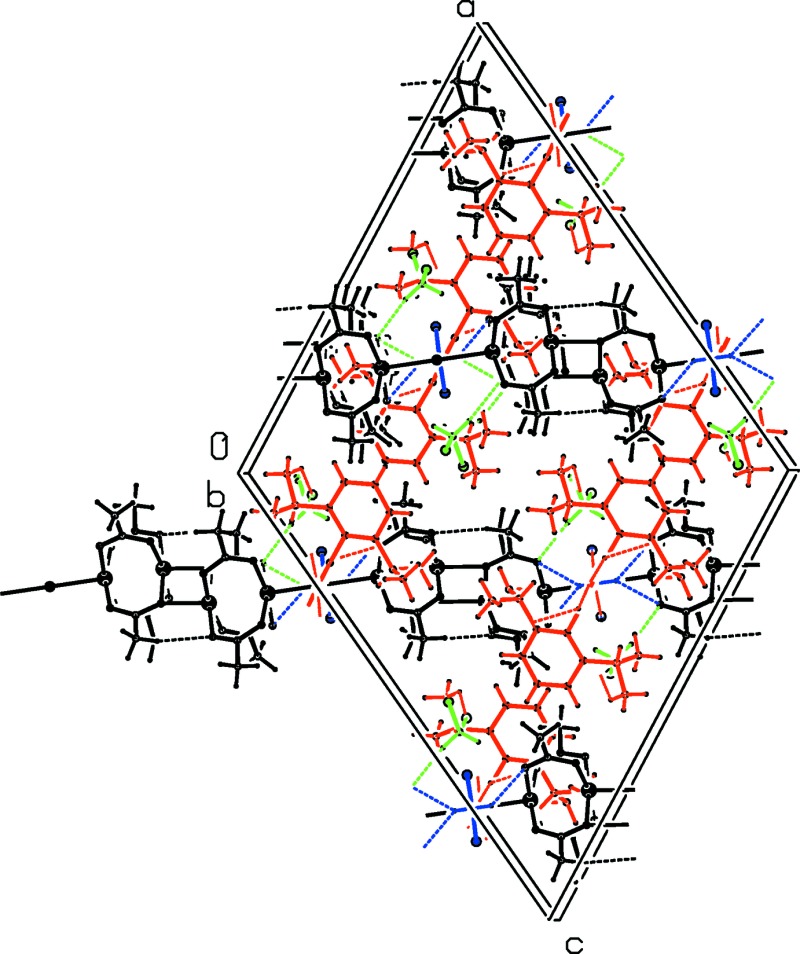
A view along the b axis of the crystal packing of title compound. Colour code: coordination polymer black, organic cation red; CH2Cl2 solvent molecules green and blue.

Click here for additional data file.x y z x y z x y z x y z x y z . DOI: 10.1107/S2056989015013675/su5152fig2.tif
A view of the tetra­nuclear paddle-wheel unit of the title polymeric compound [symmetry codes: (a) −*x*, *y*, −*z* + 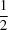; (b) −*x* + 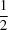, −*y* + 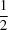, −*z* + 1; (c) *x* + 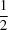, −*y* + 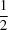, *z* + 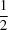; (d) −*x*, *y*, −*z* + 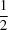; (e) −*x* + 1, *y*, −*z* + 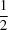].

b 2 2 . DOI: 10.1107/S2056989015013675/su5152fig3.tif
A view along the *b* axis of the crystal packing of title compound. Colour code: coordination polymer black, organic cation red; CH_2_Cl_2_ solvent mol­ecules green and blue.

CCDC reference: 999046

Additional supporting information:  crystallographic information; 3D view; checkCIF report

Enhanced figure: interactive version of Fig. 1d

## Figures and Tables

**Table 1:** Hydrogen-bond geometry (, )

*D*H*A*	*D*H	H*A*	*D* *A*	*D*H*A*
C6H6*B*O2^i^	0.98	2.43	3.368(4)	161
C21H21O1^ii^	0.95	2.54	3.344(4)	142
C22H22Cl4^iii^	0.95	2.78	3.626(5)	149
C22H22Cl4^iv^	0.95	2.78	3.626(5)	149
C23H23*A*O5^v^	0.99	2.42	3.316(5)	151
C23H23*B*O7	0.99	2.42	3.413(5)	177
C24H24*B*O5	0.99	2.42	3.303(4)	148
C24H24*B*O7	0.99	2.52	3.378(4)	145
C24H24*A*O5^v^	0.99	2.42	3.303(4)	148
C24H24*A*O7^v^	0.99	2.52	3.378(4)	145

Symmetry codes: (i) 
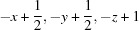
; (ii) 
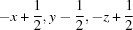
; (iii) 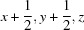; (iv) 
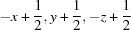
; (v) 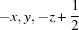.

## supplementary crystallographic information

### S1. Synthesis and crystallization

To a solution of 1,3-bis­(2,6-di-iso­propyl­phenyl)­imidazol-2-ylidine(0.22 g, 0.55 mmol) in dry toluene, at room temperature under nitro­gen, was added anhydrous copper(II)acetate (0.09g, 0.5 mmol). The reaction mixture was stirred at room temperature for 12 h and the blue coloured precipitate, identified as 1,3-bis­(2,6-di-iso­propyl­phenyl)­imidazolium copper(II) acetate, was removed by filtration. The filtrate was left to stand at 248 K in an enclosed vessel for 1 week and the precipitate was collected at the pump. Recrystallization of this solid (vapour diffusion from CH_2_Cl_2_/petrol) afforded an admixture of two crystalline products; one colourless (which proved to be 1,3-bis­(2,6-di-iso­propyl­phenyl)­imidazolium chloride) and the other, small blue block-like crystals of the title compound. Physical separation of these two crystalline compounds and further recrystallization of the blue-coloured crystals from CH_2_Cl_2_/petrol afforded crystals suitable for X-ray diffraction analysis.

### S2. Refinement

The H atoms were included in calculated positions and refined as riding atoms: C—H = 0.95 - 98 Å with U_iso_(H) = 1.5U_eq_(C) for methyl H atoms and 1.2U_eq_(C) for other H atoms.

### S3. Structural commentary

N-heterocyclic carbenes (NHCs) have been used as ancillary ligands for the preparation of transition metal based catalysts (Hopkinson *et al.*, 2014), which are very useful in organic transformations (Faulkner *et al.*, 2005; Bull *et al.*, 2008). Binuclear Cu^II^ carboxyl­ate compounds are inter­esting because of their magnetic properties (Kato *et al.*, 1964, Zhang *et al.*, 2005; Cotton *et al.*, 2000) and electrochemical behaviour (Paschke *et al.*, 2003). Herein, we report on the synthesis and crystal structure of the title copper(II) acetate coordination polymer. Here, acetate acts as a bridging bidentate chelating ligand, giving a typical paddle-wheel structure.

The asymmetric unit of the title compound, Fig. 1, is composed of a copper(II) acetate paddle-wheel complex [Cu1···Cu2 = 2.6016 Å], with atom Cu1 coordinated in the apical position by a Cl^-^ anion [Cu1—Cl1 = 2.4364 (6) Å] situated on a twofold rotation axis. Both copper(II) atoms have distorted square pyramidal co-ordination geometry with τ values of 0.04 (Addison *et al.*, 1984).

The copper(II) acetate paddle-wheel units are linked by inversion symmetry, with the apical position of the second Cu^II^ atom, Cu2, being occupied by an acetate O atom; Cu2···Cu2^i^ = 3.1944 (8) Å and Cu2···O6^i^ = 2.200 (2) Å [symmetry code: (i) - x + 1/2, - y + 1/2, - z + 1], as shown in Fig. 2. These tetra­nuclear units are bridged by the Cl atom, Cl1, coordinated to atom Cu1 and located on a twofold rotation axis, forming the one-dimensional polymer that propagates along [101]; see Fig. 2.

In the crystal, the cation and di­chloro­methane solvent molecules are linked to the coordination polymer by various C—H···O and C—H···Cl hydrogen bonds (Table 1 and Fig. 3). There are no other significant inter­molecular inter­actions present.

This structure is unique in that it possesses a halide bridge linking tetra­nuclear copper paddle-wheel units (for chloride-bridged binuclear systems, see: Chen *et al.*, 2015) and imidazolium salts inter­spersed within the crystal lattice (for imidazolium-functionalised acetate ligands, see: Suresh *et al.*, 2015).

### Crystal data

**Table:**

(C_27_H_37_N_2_)[Cu_4_(C_2_H_3_O_2_)_8_Cl]·3CH_2_Cl_2_	*F*(000) = 2880
*M**_r_* = 1406.32	*D*_x_ = 1.530 Mg m^−^^3^
Monoclinic, *C*2/*c*	Mo *K*α radiation, λ = 0.71073 Å
*a* = 22.097 (2) Å	Cell parameters from 3370 reflections
*b* = 13.146 (2) Å	θ = 2.3–24.3°
*c* = 23.607 (3) Å	µ = 1.74 mm^−^^1^
β = 117.122 (4)°	*T* = 100 K
*V* = 6103.5 (13) Å^3^	Block, blue
*Z* = 4	0.22 × 0.13 × 0.05 mm

### Data collection

**Table:**

Bruker SMART CCD area-detector diffractometer	7274 independent reflections
Radiation source: fine-focus sealed tube	5215 reflections with *I* > 2σ(*I*)
Graphite monochromator	*R*_int_ = 0.079
phi and ω scans	θ_max_ = 28.3°, θ_min_ = 1.9°
Absorption correction: multi-scan (*SADABS*; Bruker, 2001)	*h* = −29→29
*T*_min_ = 0.700, *T*_max_ = 0.918	*k* = −17→17
26111 measured reflections	*l* = −31→31

### Refinement

**Table:**

Refinement on *F*^2^	Primary atom site location: structure-invariant direct methods
Least-squares matrix: full	Secondary atom site location: difference Fourier map
*R*[*F*^2^ > 2σ(*F*^2^)] = 0.054	Hydrogen site location: inferred from neighbouring sites
*wR*(*F*^2^) = 0.098	H-atom parameters constrained
*S* = 0.98	*w* = 1/[σ^2^(*F*_o_^2^) + (0.0252*P*)^2^] where *P* = (*F*_o_^2^ + 2*F*_c_^2^)/3
7274 reflections	(Δ/σ)_max_ < 0.001
348 parameters	Δρ_max_ = 0.70 e Å^−^^3^
0 restraints	Δρ_min_ = −0.46 e Å^−^^3^

### Special details

**Table:**

Geometry. All esds (except the esd in the dihedral angle between two l.s. planes) are estimated using the full covariance matrix. The cell esds are taken into account individually in the estimation of esds in distances, angles and torsion angles; correlations between esds in cell parameters are only used when they are defined by crystal symmetry. An approximate (isotropic) treatment of cell esds is used for estimating esds involving l.s. planes.

### Fractional atomic coordinates and isotropic or equivalent isotropic displacement parameters (Å^2^)

**Table:**

	*x*	*y*	*z*	*U*_iso_*/*U*_eq_	Occ. (<1)
Cu1	0.11018 (2)	0.34481 (3)	0.34274 (2)	0.01543 (11)	
Cu2	0.22901 (2)	0.32446 (3)	0.44052 (2)	0.01720 (12)	
Cl1	0.0000	0.36569 (9)	0.2500	0.0197 (3)	
O1	0.15907 (12)	0.45167 (19)	0.32111 (12)	0.0215 (6)	
O2	0.26294 (13)	0.4197 (2)	0.39901 (12)	0.0276 (7)	
O3	0.09345 (12)	0.4383 (2)	0.39834 (12)	0.0257 (6)	
O4	0.19967 (13)	0.43484 (19)	0.47704 (12)	0.0242 (6)	
O5	0.08495 (12)	0.23045 (19)	0.38354 (12)	0.0244 (6)	
O6	0.18267 (11)	0.22502 (18)	0.47165 (11)	0.0170 (6)	
O7	0.14402 (13)	0.24162 (19)	0.30413 (12)	0.0233 (6)	
O8	0.24153 (12)	0.2136 (2)	0.39190 (12)	0.0243 (6)	
C1	0.2223 (2)	0.4655 (3)	0.34956 (18)	0.0206 (8)	
C2	0.2526 (2)	0.5414 (3)	0.32215 (19)	0.0287 (10)	
H2A	0.2161	0.5773	0.2866	0.043*	
H2B	0.2813	0.5059	0.3068	0.043*	
H2C	0.2802	0.5905	0.3551	0.043*	
C3	0.1390 (2)	0.4647 (3)	0.45198 (18)	0.0214 (9)	
C4	0.1181 (2)	0.5375 (3)	0.4893 (2)	0.0336 (11)	
H4A	0.0965	0.5974	0.4632	0.050*	
H4B	0.1583	0.5586	0.5280	0.050*	
H4C	0.0859	0.5037	0.5011	0.050*	
C5	0.12274 (18)	0.1946 (3)	0.43727 (17)	0.0182 (8)	
C6	0.09553 (18)	0.1106 (3)	0.46205 (17)	0.0231 (9)	
H6A	0.0557	0.1351	0.4660	0.035*	
H6B	0.1307	0.0889	0.5039	0.035*	
H6C	0.0823	0.0530	0.4325	0.035*	
C7	0.19844 (19)	0.1934 (3)	0.33588 (18)	0.0204 (8)	
C8	0.2120 (2)	0.1034 (3)	0.30460 (19)	0.0309 (10)	
H8A	0.2608	0.0884	0.3254	0.046*	
H8B	0.1971	0.1183	0.2595	0.046*	
H8C	0.1869	0.0444	0.3084	0.046*	
N1	0.45188 (14)	0.2152 (2)	0.25127 (13)	0.0154 (6)	
C9	0.38905 (18)	0.2530 (3)	0.24934 (18)	0.0181 (8)	
C10	0.33835 (18)	0.2839 (3)	0.19043 (18)	0.0202 (8)	
C11	0.27799 (19)	0.3182 (3)	0.18894 (18)	0.0239 (9)	
H11	0.2417	0.3396	0.1497	0.029*	
C12	0.27022 (19)	0.3217 (3)	0.24336 (19)	0.0251 (9)	
H12	0.2284	0.3447	0.2412	0.030*	
C13	0.32230 (19)	0.2922 (3)	0.30130 (19)	0.0253 (9)	
H13	0.3162	0.2966	0.3385	0.030*	
C14	0.38368 (18)	0.2560 (3)	0.30584 (18)	0.0204 (8)	
C15	0.34523 (19)	0.2799 (3)	0.12880 (18)	0.0261 (9)	
H15	0.3923	0.2559	0.1400	0.031*	
C16	0.3369 (2)	0.3836 (3)	0.09913 (19)	0.0349 (11)	
H16A	0.2901	0.4073	0.0849	0.052*	
H16B	0.3466	0.3798	0.0626	0.052*	
H16C	0.3685	0.4313	0.1306	0.052*	
C17	0.2957 (2)	0.2034 (3)	0.0823 (2)	0.0406 (12)	
H17A	0.3010	0.2023	0.0433	0.061*	
H17B	0.2491	0.2230	0.0720	0.061*	
H17C	0.3052	0.1356	0.1017	0.061*	
C18	0.44195 (19)	0.2267 (3)	0.36975 (18)	0.0252 (9)	
H18	0.4691	0.1725	0.3621	0.030*	
C19	0.4890 (2)	0.3180 (3)	0.4002 (2)	0.0407 (12)	
H19A	0.4629	0.3737	0.4060	0.061*	
H19B	0.5084	0.3406	0.3724	0.061*	
H19C	0.5257	0.2980	0.4416	0.061*	
C20	0.4180 (2)	0.1848 (3)	0.41638 (19)	0.0339 (10)	
H20A	0.3866	0.1281	0.3965	0.051*	
H20B	0.3947	0.2386	0.4277	0.051*	
H20C	0.4573	0.1608	0.4549	0.051*	
C21	0.46971 (17)	0.1151 (3)	0.25048 (16)	0.0163 (8)	
H21	0.4442	0.0570	0.2506	0.020*	
C22	0.5000	0.2738 (4)	0.2500	0.0167 (11)	
H22	0.5000	0.3460	0.2500	0.020*	
Cl2	0.08838 (7)	0.23037 (12)	0.10086 (7)	0.0627 (4)	
Cl3	0.09648 (6)	0.43818 (10)	0.14520 (6)	0.0528 (4)	
C23	0.0688 (2)	0.3145 (4)	0.1482 (2)	0.0410 (12)	
H23A	0.0190	0.3150	0.1332	0.049*	
H23B	0.0907	0.2903	0.1929	0.049*	
Cl4	0.03361 (6)	0.02638 (9)	0.20944 (6)	0.0455 (3)	
C24	0.0000	0.1022 (4)	0.2500	0.0365 (16)	
H24A	−0.0363	0.1465	0.2191	0.044*	0.5
H24B	0.0363	0.1465	0.2809	0.044*	0.5

### Atomic displacement parameters (Å^2^)

**Table:**

	*U*^11^	*U*^22^	*U*^33^	*U*^12^	*U*^13^	*U*^23^
Cu1	0.0147 (2)	0.0170 (2)	0.0125 (2)	0.00113 (18)	0.00437 (19)	0.00052 (18)
Cu2	0.0138 (2)	0.0222 (3)	0.0141 (2)	0.00123 (19)	0.00498 (19)	0.00456 (19)
Cl1	0.0180 (7)	0.0168 (6)	0.0156 (7)	0.000	0.0001 (5)	0.000
O1	0.0182 (14)	0.0239 (15)	0.0205 (15)	−0.0014 (11)	0.0069 (12)	0.0041 (11)
O2	0.0180 (14)	0.0377 (17)	0.0235 (15)	−0.0006 (12)	0.0064 (12)	0.0130 (13)
O3	0.0171 (14)	0.0358 (17)	0.0235 (15)	0.0020 (12)	0.0085 (12)	−0.0096 (13)
O4	0.0223 (15)	0.0239 (15)	0.0197 (15)	0.0014 (12)	0.0038 (12)	−0.0010 (12)
O5	0.0165 (14)	0.0302 (16)	0.0201 (15)	−0.0023 (12)	0.0027 (12)	0.0105 (12)
O6	0.0098 (12)	0.0241 (14)	0.0154 (13)	0.0002 (10)	0.0041 (11)	0.0042 (11)
O7	0.0235 (15)	0.0236 (15)	0.0196 (15)	0.0048 (12)	0.0070 (12)	−0.0040 (11)
O8	0.0207 (14)	0.0329 (16)	0.0187 (15)	0.0097 (12)	0.0085 (12)	0.0027 (12)
C1	0.026 (2)	0.019 (2)	0.024 (2)	0.0001 (17)	0.0176 (19)	−0.0020 (16)
C2	0.029 (2)	0.028 (2)	0.034 (3)	0.0015 (18)	0.019 (2)	0.0067 (19)
C3	0.030 (2)	0.020 (2)	0.021 (2)	−0.0035 (17)	0.0181 (19)	−0.0010 (16)
C4	0.038 (3)	0.037 (3)	0.033 (3)	−0.003 (2)	0.022 (2)	−0.011 (2)
C5	0.0188 (19)	0.022 (2)	0.016 (2)	0.0017 (16)	0.0097 (17)	−0.0008 (16)
C6	0.021 (2)	0.026 (2)	0.019 (2)	−0.0046 (17)	0.0058 (17)	0.0037 (17)
C7	0.027 (2)	0.020 (2)	0.024 (2)	−0.0019 (17)	0.0195 (19)	0.0021 (17)
C8	0.034 (3)	0.029 (2)	0.037 (3)	0.0050 (19)	0.023 (2)	−0.0033 (19)
N1	0.0135 (15)	0.0192 (16)	0.0143 (16)	0.0014 (12)	0.0072 (13)	−0.0009 (13)
C9	0.0156 (19)	0.0173 (19)	0.024 (2)	−0.0016 (15)	0.0115 (17)	−0.0029 (16)
C10	0.020 (2)	0.0168 (19)	0.023 (2)	0.0001 (16)	0.0092 (17)	0.0008 (16)
C11	0.020 (2)	0.026 (2)	0.024 (2)	−0.0014 (17)	0.0077 (17)	0.0007 (17)
C12	0.017 (2)	0.026 (2)	0.035 (2)	0.0037 (17)	0.0142 (18)	0.0014 (19)
C13	0.025 (2)	0.029 (2)	0.031 (2)	−0.0007 (18)	0.0198 (19)	−0.0046 (18)
C14	0.021 (2)	0.0150 (19)	0.027 (2)	−0.0003 (15)	0.0134 (18)	0.0002 (16)
C15	0.020 (2)	0.038 (3)	0.019 (2)	0.0082 (18)	0.0080 (17)	0.0059 (18)
C16	0.044 (3)	0.039 (3)	0.026 (2)	−0.008 (2)	0.020 (2)	0.001 (2)
C17	0.065 (3)	0.035 (3)	0.026 (2)	−0.005 (2)	0.024 (2)	−0.002 (2)
C18	0.022 (2)	0.039 (2)	0.020 (2)	0.0024 (18)	0.0133 (18)	−0.0001 (18)
C19	0.040 (3)	0.052 (3)	0.028 (3)	−0.008 (2)	0.013 (2)	0.001 (2)
C20	0.037 (3)	0.038 (3)	0.027 (2)	0.001 (2)	0.015 (2)	0.004 (2)
C21	0.020 (2)	0.0148 (18)	0.0162 (19)	−0.0042 (15)	0.0096 (16)	−0.0017 (15)
C22	0.019 (3)	0.016 (3)	0.017 (3)	0.000	0.010 (2)	0.000
Cl2	0.0452 (8)	0.0907 (11)	0.0573 (9)	−0.0103 (7)	0.0279 (7)	−0.0248 (8)
Cl3	0.0508 (8)	0.0583 (9)	0.0599 (9)	0.0080 (6)	0.0344 (7)	0.0196 (7)
C23	0.030 (2)	0.065 (3)	0.030 (3)	−0.007 (2)	0.015 (2)	−0.004 (2)
Cl4	0.0564 (8)	0.0313 (6)	0.0472 (8)	−0.0066 (6)	0.0223 (6)	−0.0007 (5)
C24	0.041 (4)	0.018 (3)	0.036 (4)	0.000	0.006 (3)	0.000

### Geometric parameters (Å, º)

**Table:**

Cu1—O3	1.952 (3)	C9—C14	1.393 (5)
Cu1—O7	1.963 (2)	C10—C11	1.394 (5)
Cu1—O1	1.976 (2)	C10—C15	1.531 (5)
Cu1—O5	1.997 (2)	C11—C12	1.372 (5)
Cu1—Cl1	2.4365 (5)	C11—H11	0.9500
Cu1—Cu2	2.6015 (6)	C12—C13	1.382 (5)
Cu2—O2	1.939 (3)	C12—H12	0.9500
Cu2—O4	1.944 (3)	C13—C14	1.394 (5)
Cu2—O8	1.951 (3)	C13—H13	0.9500
Cu2—O6	1.996 (2)	C14—C18	1.520 (5)
Cu2—O6^i^	2.200 (2)	C15—C16	1.506 (5)
Cl1—Cu1^ii^	2.4365 (5)	C15—C17	1.523 (5)
O1—C1	1.257 (4)	C15—H15	1.0000
O2—C1	1.255 (4)	C16—H16A	0.9800
O3—C3	1.255 (4)	C16—H16B	0.9800
O4—C3	1.257 (4)	C16—H16C	0.9800
O5—C5	1.250 (4)	C17—H17A	0.9800
O6—C5	1.262 (4)	C17—H17B	0.9800
O6—Cu2^i^	2.200 (2)	C17—H17C	0.9800
O7—C7	1.260 (4)	C18—C20	1.525 (5)
O8—C7	1.256 (4)	C18—C19	1.535 (5)
C1—C2	1.503 (5)	C18—H18	1.0000
C2—H2A	0.9800	C19—H19A	0.9800
C2—H2B	0.9800	C19—H19B	0.9800
C2—H2C	0.9800	C19—H19C	0.9800
C3—C4	1.509 (5)	C20—H20A	0.9800
C4—H4A	0.9800	C20—H20B	0.9800
C4—H4B	0.9800	C20—H20C	0.9800
C4—H4C	0.9800	C21—C21^iii^	1.349 (6)
C5—C6	1.497 (5)	C21—H21	0.9500
C6—H6A	0.9800	C22—N1^iii^	1.324 (4)
C6—H6B	0.9800	C22—H22	0.9500
C6—H6C	0.9800	Cl2—C23	1.762 (4)
C7—C8	1.496 (5)	Cl3—C23	1.750 (5)
C8—H8A	0.9800	C23—H23A	0.9900
C8—H8B	0.9800	C23—H23B	0.9900
C8—H8C	0.9800	Cl4—C24	1.764 (3)
N1—C22	1.324 (4)	C24—Cl4^ii^	1.764 (3)
N1—C21	1.376 (4)	C24—H24A	0.9900
N1—C9	1.455 (4)	C24—H24B	0.9900
C9—C10	1.390 (5)		
			
O3—Cu1—O7	167.50 (11)	H8B—C8—H8C	109.5
O3—Cu1—O1	91.12 (11)	C22—N1—C21	108.5 (3)
O7—Cu1—O1	90.14 (11)	C22—N1—C9	124.4 (3)
O3—Cu1—O5	88.26 (11)	C21—N1—C9	126.9 (3)
O7—Cu1—O5	87.30 (11)	C10—C9—C14	124.6 (3)
O1—Cu1—O5	164.93 (10)	C10—C9—N1	116.9 (3)
O3—Cu1—Cl1	96.62 (8)	C14—C9—N1	118.5 (3)
O7—Cu1—Cl1	95.55 (8)	C9—C10—C11	116.5 (3)
O1—Cu1—Cl1	97.34 (8)	C9—C10—C15	123.8 (3)
O5—Cu1—Cl1	97.69 (7)	C11—C10—C15	119.7 (3)
O3—Cu1—Cu2	83.81 (8)	C12—C11—C10	120.9 (4)
O7—Cu1—Cu2	84.08 (7)	C12—C11—H11	119.5
O1—Cu1—Cu2	81.54 (7)	C10—C11—H11	119.5
O5—Cu1—Cu2	83.42 (7)	C11—C12—C13	120.9 (4)
Cl1—Cu1—Cu2	178.81 (3)	C11—C12—H12	119.5
O2—Cu2—O4	91.50 (11)	C13—C12—H12	119.5
O2—Cu2—O8	89.64 (11)	C12—C13—C14	120.9 (4)
O4—Cu2—O8	169.67 (11)	C12—C13—H13	119.5
O2—Cu2—O6	172.08 (10)	C14—C13—H13	119.5
O4—Cu2—O6	89.70 (10)	C9—C14—C13	116.2 (4)
O8—Cu2—O6	87.79 (10)	C9—C14—C18	122.7 (3)
O2—Cu2—O6^i^	106.62 (10)	C13—C14—C18	121.1 (3)
O4—Cu2—O6^i^	97.74 (10)	C16—C15—C17	111.6 (3)
O8—Cu2—O6^i^	91.76 (10)	C16—C15—C10	111.5 (3)
O6—Cu2—O6^i^	80.96 (10)	C17—C15—C10	111.0 (3)
O2—Cu2—Cu1	87.23 (8)	C16—C15—H15	107.5
O4—Cu2—Cu1	84.89 (7)	C17—C15—H15	107.5
O8—Cu2—Cu1	84.91 (7)	C10—C15—H15	107.5
O6—Cu2—Cu1	85.09 (7)	C15—C16—H16A	109.5
O6^i^—Cu2—Cu1	165.77 (6)	C15—C16—H16B	109.5
Cu1—Cl1—Cu1^ii^	167.06 (6)	H16A—C16—H16B	109.5
C1—O1—Cu1	124.7 (2)	C15—C16—H16C	109.5
C1—O2—Cu2	120.1 (2)	H16A—C16—H16C	109.5
C3—O3—Cu1	122.8 (2)	H16B—C16—H16C	109.5
C3—O4—Cu2	121.6 (2)	C15—C17—H17A	109.5
C5—O5—Cu1	124.7 (2)	C15—C17—H17B	109.5
C5—O6—Cu2	122.3 (2)	H17A—C17—H17B	109.5
C5—O6—Cu2^i^	137.1 (2)	C15—C17—H17C	109.5
Cu2—O6—Cu2^i^	99.04 (9)	H17A—C17—H17C	109.5
C7—O7—Cu1	122.3 (2)	H17B—C17—H17C	109.5
C7—O8—Cu2	122.3 (2)	C14—C18—C20	113.1 (3)
O2—C1—O1	125.3 (3)	C14—C18—C19	110.7 (3)
O2—C1—C2	116.6 (3)	C20—C18—C19	109.2 (3)
O1—C1—C2	118.1 (3)	C14—C18—H18	107.9
C1—C2—H2A	109.5	C20—C18—H18	107.9
C1—C2—H2B	109.5	C19—C18—H18	107.9
H2A—C2—H2B	109.5	C18—C19—H19A	109.5
C1—C2—H2C	109.5	C18—C19—H19B	109.5
H2A—C2—H2C	109.5	H19A—C19—H19B	109.5
H2B—C2—H2C	109.5	C18—C19—H19C	109.5
O3—C3—O4	125.5 (4)	H19A—C19—H19C	109.5
O3—C3—C4	116.6 (3)	H19B—C19—H19C	109.5
O4—C3—C4	117.9 (3)	C18—C20—H20A	109.5
C3—C4—H4A	109.5	C18—C20—H20B	109.5
C3—C4—H4B	109.5	H20A—C20—H20B	109.5
H4A—C4—H4B	109.5	C18—C20—H20C	109.5
C3—C4—H4C	109.5	H20A—C20—H20C	109.5
H4A—C4—H4C	109.5	H20B—C20—H20C	109.5
H4B—C4—H4C	109.5	C21^iii^—C21—N1	107.02 (18)
O5—C5—O6	123.5 (3)	C21^iii^—C21—H21	126.5
O5—C5—C6	118.2 (3)	N1—C21—H21	126.5
O6—C5—C6	118.2 (3)	N1^iii^—C22—N1	108.9 (4)
C5—C6—H6A	109.5	N1^iii^—C22—H22	125.6
C5—C6—H6B	109.5	N1—C22—H22	125.6
H6A—C6—H6B	109.5	Cl3—C23—Cl2	111.4 (2)
C5—C6—H6C	109.5	Cl3—C23—H23A	109.3
H6A—C6—H6C	109.5	Cl2—C23—H23A	109.3
H6B—C6—H6C	109.5	Cl3—C23—H23B	109.3
O8—C7—O7	125.5 (3)	Cl2—C23—H23B	109.3
O8—C7—C8	117.4 (3)	H23A—C23—H23B	108.0
O7—C7—C8	117.1 (3)	Cl4—C24—Cl4^ii^	111.2 (3)
C7—C8—H8A	109.5	Cl4—C24—H24A	109.4
C7—C8—H8B	109.5	Cl4^ii^—C24—H24A	109.4
H8A—C8—H8B	109.5	Cl4—C24—H24B	109.4
C7—C8—H8C	109.5	Cl4^ii^—C24—H24B	109.4
H8A—C8—H8C	109.5	H24A—C24—H24B	108.0
			
Cu2—O2—C1—O1	−3.8 (5)	C14—C9—C10—C15	−180.0 (4)
Cu2—O2—C1—C2	177.3 (2)	N1—C9—C10—C15	0.2 (5)
Cu1—O1—C1—O2	−6.5 (5)	C9—C10—C11—C12	0.5 (6)
Cu1—O1—C1—C2	172.4 (2)	C15—C10—C11—C12	179.4 (4)
Cu1—O3—C3—O4	−0.9 (5)	C10—C11—C12—C13	0.7 (6)
Cu1—O3—C3—C4	−180.0 (3)	C11—C12—C13—C14	−1.4 (6)
Cu2—O4—C3—O3	−9.7 (5)	C10—C9—C14—C13	0.5 (6)
Cu2—O4—C3—C4	169.4 (3)	N1—C9—C14—C13	−179.6 (3)
Cu1—O5—C5—O6	−2.0 (5)	C10—C9—C14—C18	−176.6 (3)
Cu1—O5—C5—C6	178.2 (2)	N1—C9—C14—C18	3.3 (5)
Cu2—O6—C5—O5	−7.1 (5)	C12—C13—C14—C9	0.8 (5)
Cu2^i^—O6—C5—O5	−169.6 (2)	C12—C13—C14—C18	178.0 (3)
Cu2—O6—C5—C6	172.7 (2)	C9—C10—C15—C16	−120.9 (4)
Cu2^i^—O6—C5—C6	10.2 (5)	C11—C10—C15—C16	60.3 (5)
Cu2—O8—C7—O7	1.6 (5)	C9—C10—C15—C17	114.0 (4)
Cu2—O8—C7—C8	−177.0 (2)	C11—C10—C15—C17	−64.7 (5)
Cu1—O7—C7—O8	−10.2 (5)	C9—C14—C18—C20	−151.1 (4)
Cu1—O7—C7—C8	168.4 (2)	C13—C14—C18—C20	31.8 (5)
C22—N1—C9—C10	77.5 (4)	C9—C14—C18—C19	86.0 (4)
C21—N1—C9—C10	−98.0 (4)	C13—C14—C18—C19	−91.0 (4)
C22—N1—C9—C14	−102.4 (4)	C22—N1—C21—C21^iii^	0.8 (5)
C21—N1—C9—C14	82.2 (5)	C9—N1—C21—C21^iii^	176.8 (4)
C14—C9—C10—C11	−1.2 (6)	C21—N1—C22—N1^iii^	−0.29 (17)
N1—C9—C10—C11	178.9 (3)	C9—N1—C22—N1^iii^	−176.4 (4)

Symmetry codes: (i) −*x*+1/2, −*y*+1/2, −*z*+1; (ii) −*x*, *y*, −*z*+1/2; (iii) −*x*+1, *y*, −*z*+1/2.

### Hydrogen-bond geometry (Å, º)

**Table:**

*D*—H···*A*	*D*—H	H···*A*	*D*···*A*	*D*—H···*A*
C6—H6*B*···O2^i^	0.98	2.43	3.368 (4)	161
C21—H21···O1^iv^	0.95	2.54	3.344 (4)	142
C22—H22···Cl4^v^	0.95	2.78	3.626 (5)	149
C22—H22···Cl4^vi^	0.95	2.78	3.626 (5)	149
C23—H23*A*···O5^ii^	0.99	2.42	3.316 (5)	151
C23—H23*B*···O7	0.99	2.42	3.413 (5)	177
C24—H24*B*···O5	0.99	2.42	3.303 (4)	148
C24—H24*B*···O7	0.99	2.52	3.378 (4)	145
C24—H24*A*···O5^ii^	0.99	2.42	3.303 (4)	148
C24—H24*A*···O7^ii^	0.99	2.52	3.378 (4)	145

Symmetry codes: (i) −*x*+1/2, −*y*+1/2, −*z*+1; (ii) −*x*, *y*, −*z*+1/2; (iv) −*x*+1/2, *y*−1/2, −*z*+1/2; (v) *x*+1/2, *y*+1/2, *z*; (vi) −*x*+1/2, *y*+1/2, −*z*+1/2.

## References

[bb1] Ackermann, H., Neumüller, B. & Dehnicke, K. (2000). *Z. Anorg. Allg. Chem.* **626**, 1712–1714.

[bb2] Addison, A. W., Rao, T. N., Reedijk, J., van Rijn, J. & Verschoor, G. C. (1984). *J. Chem. Soc. Dalton Trans.* pp. 1349–1356.

[bb3] Bruker (2001). *SMART*, *SAINT* and *SADABS*. Bruker AXS Inc., Madison, Wisconsin, USA.

[bb4] Bull, J. A., Hutchings, M. G., Luján, C. & Quayle, P. (2008). *Tetrahedron Lett.* **49**, 1352–1356.

[bb5] Chen, D.-M., Ma, J.-G. & Cheng, P. (2015). *Dalton Trans.* **44**, 8926–8931. 10.1039/c5dt00994d 25873315

[bb6] Cotton, F. A., Dikarev, E. V. & Petrukhina, M. A. (2000). *Inorg. Chem.* **39**, 6072–6079. 10.1021/ic000663h 11151506

[bb7] Faulkner, J., Edlin, C. D., Fengas, D., Preece, I., Quayle, P. & Richards, S. N. (2005). *Tetrahedron Lett.* **46**, 2381–2385.

[bb8] Hopkinson, M. N., Richter, C., Schedler, M. & Glorius, F. (2014). *Nature*, **510**, 485–496. 10.1038/nature13384 24965649

[bb9] Kato, M., Jonassen, H. B. & Fanning, J. C. (1964). *Chem. Rev.* **64**, 99–128.

[bb10] Meester, P. de, Fletcher, S. R. & Skapski, A. C. (1973). *J. Chem. Soc. Dalton Trans.* pp. 2575–2578.

[bb11] Paschke, R., Liebsch, S., Tschierske, C., Oakley, M. A. & Sinn, E. (2003). *Inorg. Chem.* **42**, 8230–8240. 10.1021/ic0301021 14658873

[bb12] Sheldrick, G. M. (2008). *Acta Cryst.* A**64**, 112–122. 10.1107/S0108767307043930 18156677

[bb13] Sheldrick, G. M. (2015). *Acta Cryst.* C**71**, 3–8.

[bb14] Spek, A. L. (2009). *Acta Cryst.* D**65**, 148–155. 10.1107/S090744490804362X PMC2631630 19171970

[bb15] Suresh, P., Babu, C. N. & Prabusankar, G. (2015). *Polyhedron*, **89**, 322–329.

[bb16] Zhang, J., Hubert-Pfalzgraf, L. G. & Luneau, D. (2005). *Polyhedron*, **24**, 1185–1195.

