# Supplementary figures and images for: Crystal structure of catena-poly[hemi[1,3-bis­(2,6-diisoprop­ylphenyl)imidazolium] [[μ3-acetato-κ3 O:O:O′-tri-μ2-acetato-κ6 O:O′-dicopper(II)(Cu—Cu)]-μ-chlorido] di­chloro­methane sesqui­solvate]
Source: Acta Crystallogr E Crystallogr Commun. 2015 Jul 25;71(Pt 8):m148–9. doi: 10.1107/S2056989015013675 (PMC4571378; doi:10.1107/S2056989015013675)

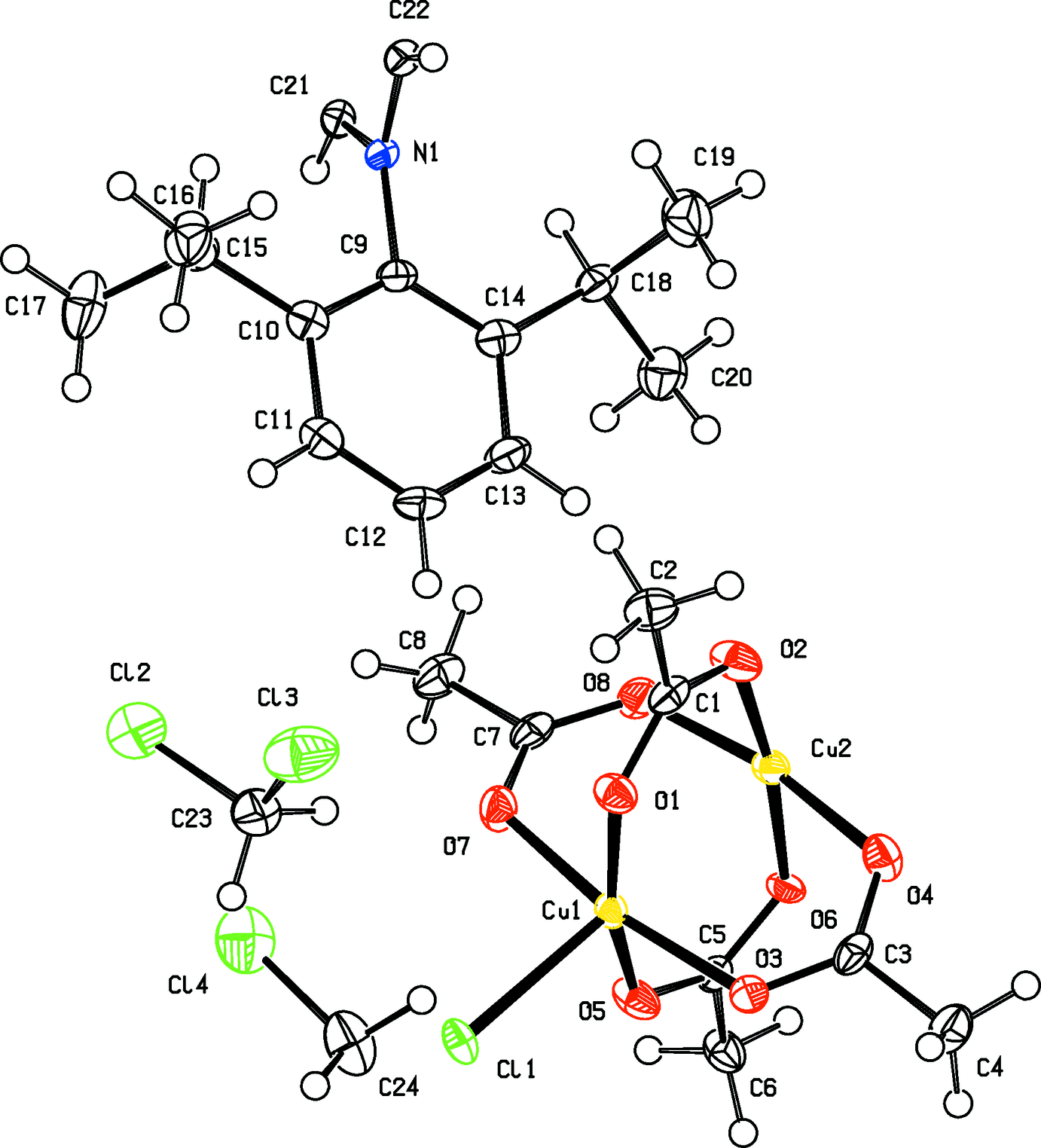

Supplement: Supplementary file 3 [file e-71-0m148-fig1.tif]

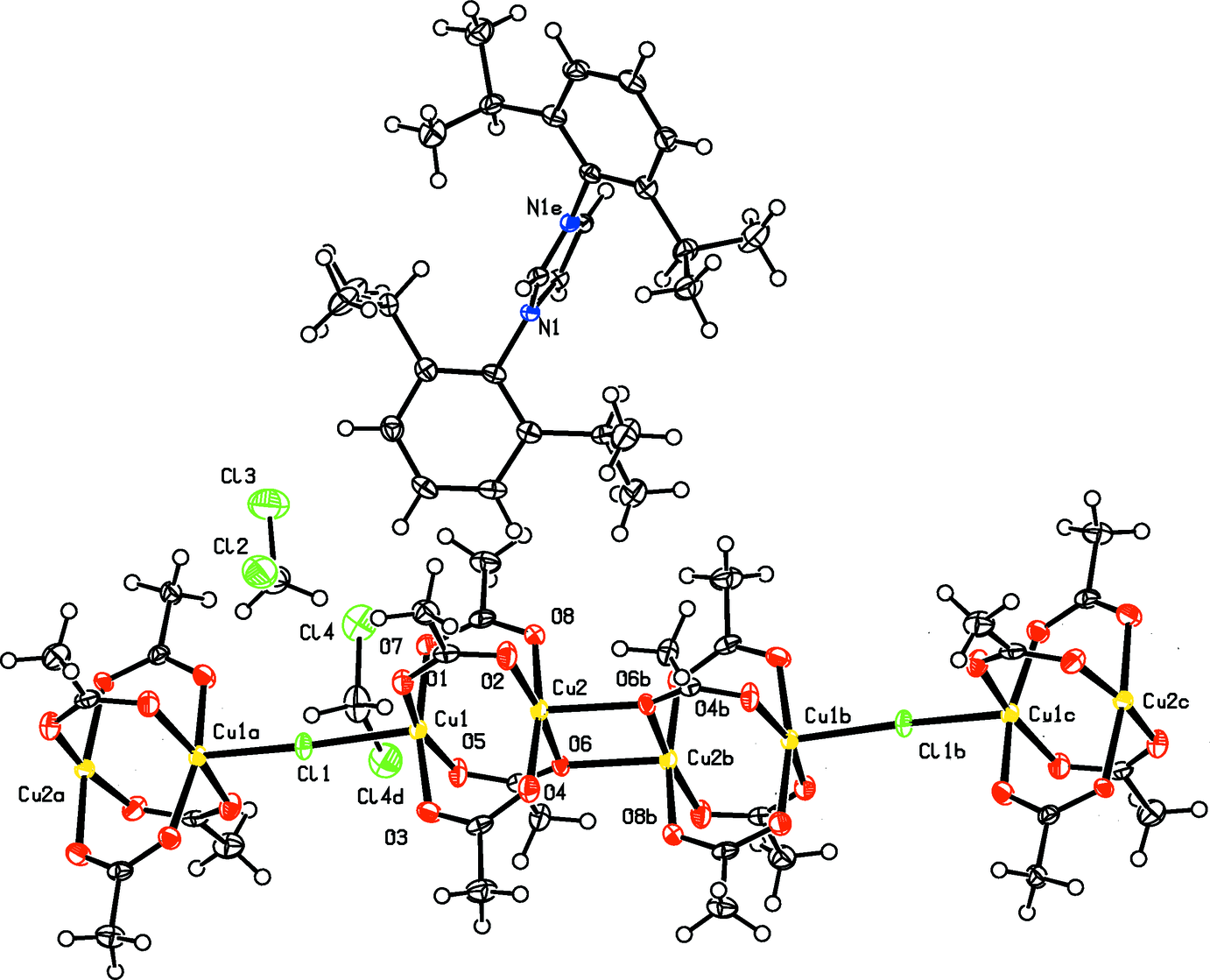

Supplement: Supplementary file 4 [file e-71-0m148-fig2.tif]

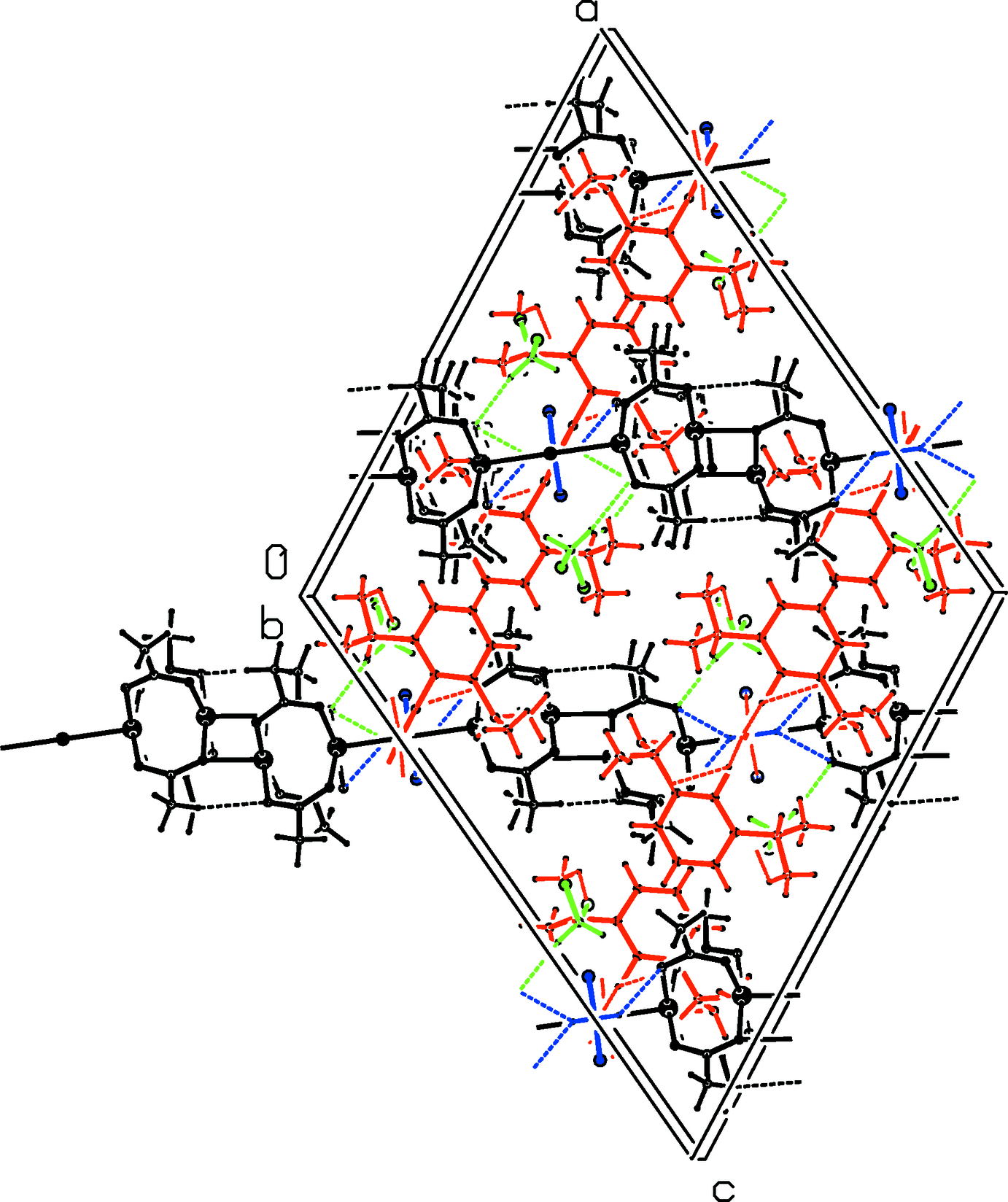

Supplement: Supplementary file 5 [file e-71-0m148-fig3.tif]
